# Fine particulate matter air pollution and the mortality of children under five: a multilevel analysis of the Ethiopian Demographic and Health Survey of 2016

**DOI:** 10.3389/fpubh.2023.1090405

**Published:** 2023-06-01

**Authors:** Ashenafie Bereded Shiferaw, Abera Kumie, Worku Tefera

**Affiliations:** ^1^Department of Social and Public Health, College of Health Sciences, Debre Tabor University, Debre Tabor, Ethiopia; ^2^Department of Environmental and Behavioral Medicine, School of Public Health, College of Health Sciences, Addis Ababa University, Addis Ababa, Ethiopia

**Keywords:** particulate matter <2.5 μm (PM) 2.5, under-five children, air pollution, sub-Sharan Africa, Ethiopia, outdoor air pollution, Demographic Health Survey (DHS), Atmospheric Composition Analysis Group (ACAG)

## Abstract

**Background:**

Every year, polluted air is costing the globe 543,000 deaths of children under five. The particulate matter below 2.5 μm diameter (PM_2.5_) is a part of air pollution that has adverse effects on children’s health. In Ethiopia, the effect of ambient PM_2.5_ is least explored. This study aimed to assess the association between PM_2.5_ and under-five mortality in Ethiopia.

**Methods:**

The study used the data from the Ethiopian Demographic Health Surveys conducted in 2016, collected between January 18 and June 27. All children under five who had data on child mortality and location coordinates were included in the study. Exposure to ambient PM_2.5_ concentration was a satellite-based estimate by the Atmospheric Composition Analysis Group at Washington and Dalhousie University, in the United States and Canada, respectively. Annual mean pollution levels and mortality datasets were matched by children’s geographical location and dates of birth, death, and interview. The relationship between ambient PM_2.5_ and under-five mortality was determined by a multilevel multivariable logistic regression on R software. The statistical analyses were two-sided at a 95% confidence interval.

**Results:**

The study addressed 10,452 children with the proportion of under-five mortality being 5.4% (95% CI 5.0–6.8%). The estimated lifetime annual mean exposure of ambient total PM_2.5_ was 20.1 ± 3.3 μgm^−3^. A 10-unit increase in the lifetime annual mean ambient total PM_2.5_ was associated with 2.29 [95% CI 1.44, 3.65] times more odds of under-five mortality after adjusting for other variables.

**Conclusion:**

Children under five are exposed to higher levels of ambient PM_2.5_ concentration, exceeding the limit set by the World Health Organization. Ambient PM_2.5_ is significantly associated with under-five mortality, adjusting for other variables. Strong measures need to be taken to reduce air pollution.

## Introduction

Every year, ambient air pollution is costing the globe around 3 million premature deaths while contributing to climate change and adverse economic impacts. The magnitude of pollution and its impact is higher in low- and middle-income countries (LMICs) (1). Ambient air pollution affects the entire population and all age groups, with children and the older adult being more susceptible (2).

Globally, among children under five, 543,000 deaths are attributed to both polluted indoor and outdoor air (3). Low nasal filtration, high ventilation rates, and higher metabolic rates in children might have contributed to a higher tendency of these pollutants to be deposited in them, consequently, affecting the lungs, renal and hepatic functions (4), and growth of the lungs (5). The burden is higher in Africa due to the higher consumption of polluting fuels, mainly biomass fuels (3).

Fine particulate matter of 2.5 μm diameter (PM_2.5_) is one of the indicators of ambient air quality that is monitored (6, 7). Particulate matter (PM) is suspended particles in the ambient and indoor air that constitute both solid and liquid particles of any kind (8). Among children, short-term PM_2.5_ exposure has been associated with pneumonia (9, 10) and upper and lower respiratory tract infection (ULRI) (10). PM_2.5_ is also associated with an increase in viral and bacterial load (11), cough, wheezing, and lower respiratory infection in children (12). Pneumonia (3, 13–15) and acute respiratory illness are the leading causes of under-five mortality in the world (3). A longer time of exposure was associated with symptoms of cough, convulsion, or fever (16) and higher rates of all-cause under-five mortality in Nairobi (16) and western and central Africa (17) and infant mortality in sub-Saharan Africa (18). Most air pollution-related health impact assessments are based on long-term exposures including annual mean concentrations (19–21).

However, previous studies showing the strong relationship between ambient PM_2.5_ and its detrimental human health effect have largely been conducted in developed nations; most are in North America and Europe. In developed nations, there is a relatively low air pollution status (22, 23) which might contribute to the small amount of mortality compared to developing countries. Hence, pooled estimates on the impact magnitude of ambient PM_2.5_ from major studies conducted in the global north might be underestimated (18, 24, 25). This was apparent in the pooled estimates from a meta-analysis of studies from five WHO regions—estimates of PM_2.5_ and all-cause mortality association showed 0.25 to 2.08% variations across the regions (22), China (23), and at the global level (25). In Africa, only a few studies have investigated and found that long-term exposure to ambient PM_2.5_ was associated with child mortality (16–18, 26). Thus, further study helps to better understand the degree of the effect of ambient PM_2.5_ in Africa.

Secondly, though under-five mortality has largely reduced since 2000 in sub-Saharan Africa, it remains the highest in need of urgent attention—75.8 deaths per 1,000 live births in 2019 (27, 28). Ethiopia is among the sub-Saharan African countries where the under-five mortality rate declined to 59 deaths per 1,000 live births in 2019 (29) from 116 deaths per 1,000 live births in 2006 (30, 31). The reduction was achieved due to improvements in sanitation, nutrition, and access to maternal and child health services. The 59 deaths per 1,000 live births is much higher than the aim or goal intended to be achieved, which is 25 deaths per 1,000 live births (32). The role of PM_2.5_ in the death toll of under-five mortality is still a subject of debate. Thus, further studies will help to better explain the role of PM_2.5_ on under-five mortality and reduce deaths (18).

Thirdly, due to the country’s insubstantial monitoring stations, apparatus, and competency of technicians, there are minimal inquiries into ambient PM_2.5_ and its adverse impact on health. Hence, there is a clear knowledge gap about ambient PM_2.5_ and its relationship with under-five mortality in Ethiopia. To the best of our knowledge, this paper is the first in Ethiopia to investigate ambient PM_2.5_ and its association with under-five mortality by controlling for individual characteristics.

## Materials and methods

### Study area and study design

Ethiopia is the largest landlocked country, with an estimated population of 110 million in the horn of Africa, located between 3°24’ and 14°15’ North and 33°00’ and 48°00’ East. It has nine regions and two cities divided for administrative purposes (33). The Ethiopian Demographic Health Survey (EDHS) followed a two-stage community-based cross-sectional study design. EDHS 2016 was collected from January 18 to June 27, 2016 (30).

### Source and study population

The source population of the study was all children under five born 5 years before the survey period (January 2011 to June 2016) in Ethiopia. While all children under five born 5 years before the survey period in the selected Enumeration Areas (EAs) were the study population.

### The inclusion criteria

All children under five born 5 years before the survey period in the selected households whose mortality data and cluster’s Global Positioning System (GPS) coordinates were recorded in the EDHS dataset were included in this study. Children without mortality and geographical location coordinates information were excluded.

### Sample size

**Figure 1:**
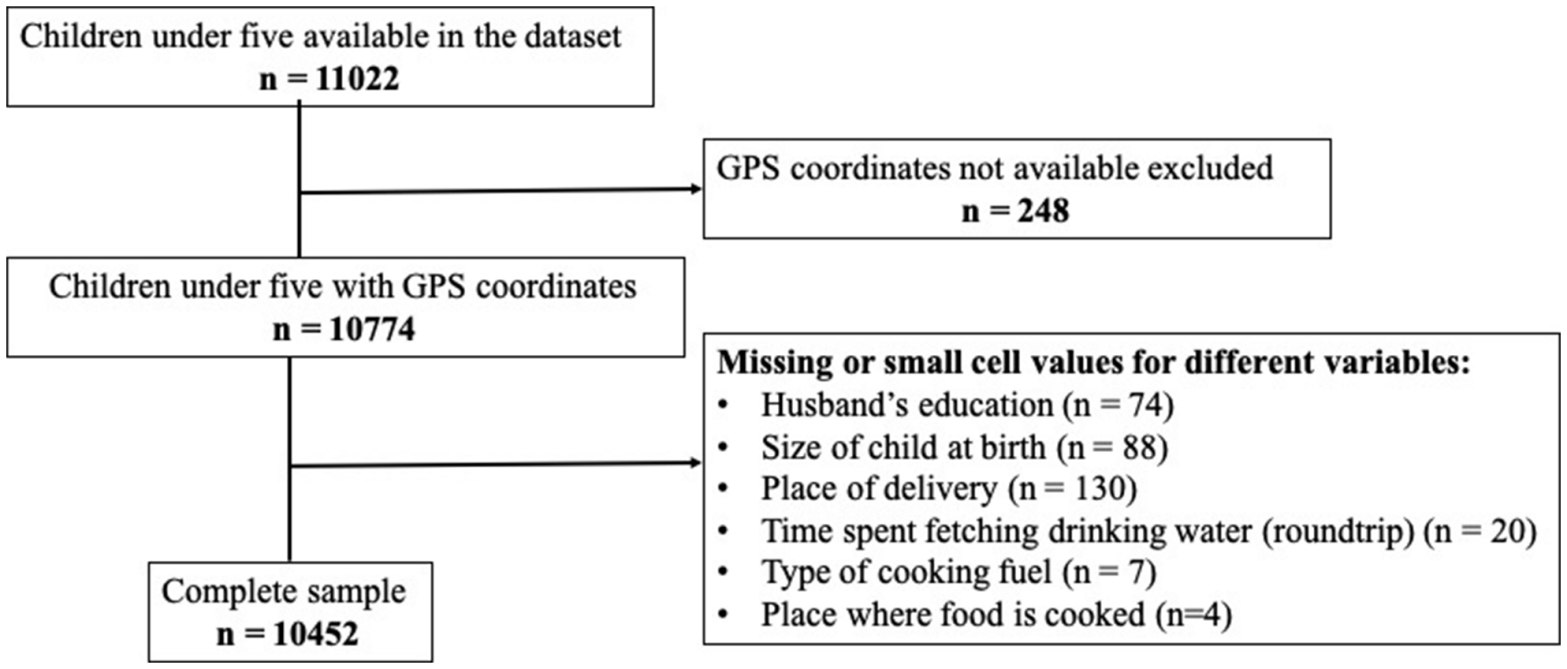
Flow chart of the inclusion of observations in the dataset for each step of this study sample analysis.

The EDHS 2016 selected 645 EAs (202 urban and 443 rural EAs). Information was collected for 10,641 (11,022 weighted) children under five (30). After removing clusters with no location coordinates and data cleaning, a total of 9,856 (10,452 weighted) children under five were included in this study (Figure 1).

Ambient PM_2.5_ concentration data derived from satellites were available across all geographical spaces in the study area.

### Sampling procedures

The EDHS collects samples that are representative of both the regional and national levels as well as urban and rural areas. Its data is also to be comparable across different nations through standardized questionnaires. Households were selected after a list of first-level administrative units and their list of enumeration areas (EAs) was provided. We selected an equal number of households (28 households from each EA) from the registered list of households in the EAs using the systematic probability method. Information from all women aged 15–49 years (reproductive-aged women) was collected in all selected households. The women provided information about their children who were under five if they had any. The sampling, methodology, and procedure details have been found published elsewhere (30).

### Data collection tools and procedures

The data of children under five needed for this study were retrieved from the Demographic Health Survey (DHS) website.^1^ The dataset with filenames starting with ETBR (birth record), ETHR (a household record), ETPR (an individual record), and ETGE (geographical location) was downloaded. The EDHS 2016 interviewers used tablets (personal digital assistants) to enter the responses of study participants. The 2016 EDHS also used a standardized questionnaire by translating the questions into local languages and back to the original language. Interviewers were also calibrated by providing training and frequent supervision before and during data collection, respectively (30).

### Dependent variable

Under-five mortality also known as death before the age of 60 months was the dependent variable, with dichotomous values of “dead” or “alive.”

### Exposure variable

Exposure to lifetime annual mean total PM_2.5_ in μgm^−3^ (microgram per cubic meter) atmospheric air is the main independent variable. In this study, “PM_2.5_ is a mixture of solid and liquid particles suspended in the ambient air” and its “mass concentration of particles diameter is less than 2.5 μm.”

### Controlled variables

The socio-demographic (mother’s educational status, age of the mother, household wealth index, mother’s employment status, mother’s marital status, husband’s occupational status, husband’s educational level, and family size), individual/child-related (sex of the child, birth order, child age, size of the child at birth, plurality of a child, and place of birth), environmental-related [source of drinking water (categorized into “improved” and “unimproved”), type of toilet/latrine facility (“improved” and “unimproved”)—categorized according to the updated joint monitoring program (JMP),^2^ presence of any kind of drinking water treatment in the household (“Yes” or “No”), type of cooking fuel (recorded as “clean fuels” if the fuel is liquefied petroleum gas (LPG), natural gas, biogas, or electricity; other kinds of cooking fuel are in the category of “solid fuels”), and place where food is cooked (“not inside the house,” “inside the house in a separate room,” or “inside the house not in a separate room”)], and community-level (region and residence) variables were controlled.

### Data on children’s ambient total PM_2.5_ exposure

In Ethiopia, there is a limited air quality monitoring system (22, 34). Accordingly, in the absence of air quality monitoring systems (ground-based PM_2.5_ concentration data), satellite-based PM_2.5_ concentration estimates across the globe surface have been employed (22, 35). This study, therefore, used satellite-based PM_2.5_ concentration estimated by the Washington and Dalhousie Universities, Atmospheric Composition Analysis Group (ACAG). The ACAG at the universities produces estimates of PM_2.5_ across the globe, including Ethiopia, at 0.01° × 0.01° spatial resolution. The group estimates the ground-level PM_2.5_ from the year 1998 to 2019 using satellite remote sensing (NASA’s MODIS, MISR, and SeaWiFS) AOD retrievals and combining it with the chemical transport model (from GEOS-Chem). They also validate their estimate with land-based PM_2.5_ monitoring systems by employing Geographically Weighted Regression (GWR). The detailed estimation technique is published elsewhere (35). The V4.GL.02 version dataset was used, and it is available publicly for free and can be downloaded from https://sites.wustl.edu/acag/datasets/surface-pm2-5/. The annual mean PM_2.5_ concentration for the years from 2011 to 2016 was extracted from the downloaded ArcGIS-compatible raster datasets.

The lifetime annual mean total PM_2.5_ concentration exposure for each child was determined. For dead children, the lifetime annual mean total PM_2.5_ concentration was the exposure period of all the months up until death; for alive children, the lifetime annual mean total PM_2.5_ concentration was the exposure period up until the month EDHS 2016 data was collected.

The geographical coordinates of EAs’ were matched with the average annual total PM_2.5_ concentration data. The DHS randomly displaced the EA’s geographical coordinates up to 2 km in urban and 5–10 km in rural areas to protect the respondents’ privacy. As a result, the average annual total PM_2.5_ within a circular radius of 3.3 km in urban areas and 6.7 km in rural areas was taken for each cluster. The month and year of the child’s date of death, birth, and interview were matched with the EA’s average annual total PM_2.5_ concentration. A weighted total PM_2.5_ exposure was taken if the exposure period falls within two or more different years—the weighting procedure has been described in detail in previous studies (18, 26).

### Data management procedures

The DHS datasets were merged using common/identifier variables (identification (ID), cluster, household, line, and birth number). R software version 3.6.3 was used to merge, recode, clean, and run descriptive and multilevel regression models while matching the geographical locations, raster clip, raster residual analysis, and sample raster values were performed using ArcGIS software version 10.7.

### Data analysis

Before any analysis, the sample was weighted for the complex survey design (36), the variables were described, and sensitivity analysis was done. Sensitivity analysis is a method used to check the influence of the changes in the values of the independent variables (i.e., of missing values) on the bivariate relationship of the independent and dependent variables (37). Proportions across sub-groups and mean and standard deviations (SD) for continuous variables were used to summarize the variables.

A separate bivariate logistic regression model was built for each controlled variable and the lifetime annual mean PM_2.5_ exposure to assess their association with under-five mortality. Variables that showed a significance level of 15% were considered for the multilevel multivariate model. The degree of variation of under-five mortality among clusters was determined using two methods; the first method used was to compare the statistically significant difference between a null logistic regression and a null multilevel logistic regression model using EAs as random effect intercept. The second method used was to describe the heterogeneity of under-five mortality among clusters using interclass correlation (ICC). The ICC is “*a measure of the proportion of variation in the outcome or under-five mortality that occurs between groups or clusters* versus *the total variation present*” (38). In the multilevel multivariate logistic regression model, controlled variables have been added to the model step by step. Socio-demographic, child-related, environmental-related, and community-level variables were added to the model, respectively. Model assumptions were checked if they were met. The final model was selected by a backward stepwise variable selection technique. At each step, variables with a significance level of 15% and variance inflation factor (VIF) > 3 (39) were removed from the model. The selected final model had the highest log-likelihood. All statistical tests computed were two-sided tests at a 95% confidence interval.

### Data quality and processing

The EDHS used re-interviewing households, checking one to two interviewers’ questionnaires per data collector, reviewing periodical field control table tools, dual entry of information by two different persons, and cleaning of missed values to ensure the quality of data (30). In this study, a careful dataset merging using dataset “identifier” or common variables, data quality parameters (36), and defensive codding while cleaning the data was done. Care was also taken in the choice of the coordinate reference system (CRS) and statistical packages (40).

### Ethics approval and consent to participate

Permission was gotten from the DHS archive and geographic database to use the DHS data. The global estimate of annual mean PM2.5 concentration was made accessible to the public by the ACAG and was used for free. Furthermore, the study was approved by the College of Health Sciences of Addis Ababa University Ethical Review Committee with a reference number SPH/1119/13.

## Results

### Characteristics of respondents

The size of the sample included in this study analysis was 10,452. The overall children’s mothers’ age mean (SD) was 29.6 ± 6.6. Children’s mothers between 25 and 29 years old constituted the majority, 29.9%. Most of the children’s mothers were married or living with a partner (94.9%), not working (55.5%), with no education status (65.6%), and had the poorest wealth index (23.2%). The study participants with a man as the head of the household (86.3%), a family size above three (51.4%), a husband with no education (47.8%), and a working husband (92.6%) were also the majority (Table 1).

**Table 1:** Socio-demographic factors of the study participants EDHS 2016, *n* = 10,452.

	Total population		Under-five mortality		
	*n*	%	Dead (%)	Alive (%)	P**
Socio-demographic factors					
**Age of the mother**					0.31
15–24	2,271	21.7	5.9	94.1	
25–29	3,192	30.5	5.8	94.2	
30–34	2,372	22.7	5.1	94.9	
≥35	2,617	25.0	6.4	93.6	
**Mother’s education**					<0.001*
No education	6,858	65.6	6.5	93.5	
Primary	2,823	27.0	5.0	95.0	
Secondary and above	771	7.4	3.8	96.2	
**Mother’s current marital status**					0.88
Not in a union/not living with a partner	535	5.1	6.0	94.0	
In a union/living with a partner	9,917	94.9	5.8	94.2	
**Mother’s employment status**					0.36
Not working	5,806	55.5	6.0	94.0	
Working	4,646	44.5	5.6	94.4	
**Wealth index**					<0.001*
Poorest	2,428	23.2	7.2	92.8	
Poorer	2,373	22.7	6.1	93.9	
Middle	2,173	20.8	5.3	94.7	
Richer	1926	18.4	6.1	93.9	
Richest	1,552	14.9	3.3	96.7	
**Husband’s education**					0.003*
No education	4,739	47.8	6.6	93.4	
Primary	3,945	39.8	5.5	94.5	
Secondary and above	1,233	12.4	4.4	95.6	
**Husband’s employment status**					0.15
Not working	737	7.4	6.9	93.1	
Working	9,180	92.6	5.7	94.7	
**Family size**					<0.001*
≤three	5,078	48.6	6.9	93.1	
Above three	5,374	51.4	4.7	95.3	
**Sex of household head**					0.47
Female	1,434	13.7	5.5	94.5	
Male	9,018	86.3	5.9	94.1	

**A bivariate logistic regression. *Used in the multilevel multivariate analysis.

The total sample size proportion of under-five mortality was 5.4% (95% CI 5.0–5.8%). According to respondents, 52% of the children under five were boys, 41.6% had average birth weight, 2.5% were twins, and 26.7% were born in institutions. Children’s age mean ± SD in months was 27.8 ± 18.7, the category ≥24 months constitutes 54.5% (Table 2).

**Table 2:** Child-related factors of the study participants EDHS 2016, *n* = 10,452.

	Total population		Under-five mortality		
	*n*	%	Dead (%)	Alive (%)	P**
Child/individual factors					
**Sex of the child**					<0.001*
Female	5,018	48.0	4.9	95.1	
Male	5,434	52.0	6.7	93.3	
**Size of the child at birth**					<0.001*
Very large	1891	18.1	6.8	93.2	
Large	1,466	14.0	4.8	95.2	
Average	4,351	41.6	5.0	95.0	
Small	1,059	10.1	5.9	94.1	
Very small	1,685	16.1	7.8	92.2	
**Child age (in months)**					<0.001*
≤12	2,823	27.0	18.3	81.7	
12–24	1934	18.5	3.6	96.4	
>24	5,695	54.5	0.5	99.5	
**Birth order**					0.06*
Third and below	5,121	49.0	5.4	94.6	
Fourth and above	5,331	51.0	6.3	93.7	
**The plurality of a child**					<0.001*
Yes	265	2.5	20.4	79.6	
No	10,187	97.5	5.4	94.6	
**Place of delivery**					<0.001*
Home	7,662	73.3	6.7	93.3	
Institution	2,790	26.7	4.0	96.0	

**A bivariate logistic regression. *Used in the multilevel multivariate analysis.

The lifetime annual mean satellite-based ambient PM_2.5_ exposure among children under five was 20.1 ± 3.3 μg m^−3^. The majority (67.2%) of households collect their drinking water within ≤30 min roundtrip distance. The proportion of households with improved drinking water sources, improved toilet facilities, and clean fuel was 56.8, 5.1, and 3.4%, respectively. Most (80.9%) of the respondents were residing in rural areas (Table 3).

**Table 3:** Environmental-related and community-level factors of the study participants EDHS 2016, *n* = 10,452.

	Total population		Under-five mortality		
	*n*	%	Dead (%)	Alive (%)	P**
Environmental factors					
**Ambient PM**_**2.5**_ **(mean ± SD)**	20.1 ± 3.3	21.7 ± 5.2	20.0 ± 3.3	*0.001*	20.1 ± 3.3
**Source of drinking water**					0.009*
Improved	5,940	56.8	5.3	94.7	
Unimproved	4,512	43.2	6.6	93.4	
**Time to fetch (roundtrip in minutes)**					0.02*
≤30	7,018	67.2	5.4	94.6	
>30	3,434	32.8	6.6	93.4	
**Any kind of household water treatment**					0.31
Yes	881	8.4	5.1	94.9	
No	9,571	91.6	5.9	94.1	
**Type of toilet facility**					<0.01
Improved	533	5.1	3.1	96.9	
Unimproved	9,919	94.9	6.1	93.9	
**Type of cooking fuel**					<0.001*
Clean fuel	352	3.4	2.3	97.7	
Solid fuel	10,100	96.6	6.0	94.0	
**The place where food is cooked**					<0.001*
Outside the house	5,978	57.2	5.1	94.9	
Inside but in a separate room	711	6.8	5.0	95.0	
Inside but not in a separate room	3,763	36.0	7.6	92.4	
Community-level variables					
Type of place of residence					<0.001*
Urban	1,179	11.3	3.1	96.9	
Rural	9,273	88.7	6.5	93.5	
Region					<0.001*
Tigray	679	6.5	3.8	96.2	
Afar	112	1.1	8.7	91.3	
Amhara	2009	19.2	4.8	95.2	
Oromia	4,628	44.3	5.5	94.5	
Somali	351	3.4	6.4	93.6	
Benishangul	115	1.1	7.4	92.6	
SNNPR	2,227	21.3	5.4	94.6	
Gambela	25	0.2	6.1	93.9	
Harari	25	0.2	6.9	93.1	
Addis Ababa	238	2.3	2.7	97.3	
Dire Dawa	43	0.4	5.5	94.5	

**A bivariate logistic regression. *Used in the multilevel multivariate analysis.

### Determining the association between ambient PM_2.5_ concentration and under-five mortality in Ethiopia

In the bivariate logistic model of lifetime annual mean PM_2.5_ and under-five mortality, the lifetime annual mean PM_2.5_ showed a significant association with under-five mortality (*p* = 0.001) (Table 3).

### Fitting multilevel multivariate logistic regression model

There was a significant difference between the null multilevel logistic regression model, EAs being the random effect intercept, and the empty logistic regression model (value of *p* <0.001). The ICC and the random effect intercept variance of the null model were 10.0% and 0.34, respectively.

The final model selected based on the criteria set prior to analysis was Model 4, as seen in Table 4. In the final model, a 10-unit increase in lifetime annual mean exposure to ambient PM_2.5_ was associated with 2.29 [95%CI 1.44–3.65] times more odds of being dead, adjusting for all other variables (Table 4).

**Table 4:** Multivariable multilevel logistic regression analysis of PM_2.5_ and other factors associated with under-five mortality, EDHS 2016, *n* = 10,452.

		AOR** 95% CI	AOR*** 95% CI	AOR**** 95% CI
	Under-five mortality (%)	Model 2	Model 3	Model 4
PM_2.5_ (μg m^−3^) (mean ± SD)^a^*	21.7 ± 5.2	1.34 (1.07, 1.66)*	1.33 (1.07, 1.66)*	2.29 (1.44, 3.65)*
Mother’s education				
No education	6.5	1.58 (1.08, 2.31)*	1.74 (1.15, 2.62)*	1.65 (1.08, 2.54)*
Primary	5.0	1.15 (0.78, 1.69)	1.15 (0.78, 1.73)	1.10 (0.72, 1.67)
Secondary and above	3.8	(Reference)		
Wealth index				
Poorest	7.2	2.08 (1.49, 2.89)*	1.24 (0.85, 1.80)	0.80 (0.48, 1.33)
Poorer	6.1	1.84 (1.29, 2.63)*	1.18 (0.79, 1.74)	0.88 (0.53, 1.45)
Middle	5.3	1.59 (1.09, 2.32)*	1.10 (0.73, 1.66)	0.86 (0.52, 1.44)
Richer	6.1	1.95 (1.35, 2.82)*	1.29 (0.86, 1.93)	1.12 (0.68, 1.85)
Richest	3.3	(Reference)	(Reference)	(Reference)
Family size				
≤three	6.9	(Reference)	(Reference)	(Reference)
Above three	4.7	0.55 (0.46, 0.66)*	0.57 (0.47, 0.70)*	0.58 (0.47, 0.71)*
Sex of the child				
Female	4.9		0.67 (0.55, 0.81)*	0.66 (0.55, 0.80)*
Male	6.7		(Reference)	(Reference)
Size of the child at birth				
Very large	6.8		1.43 (1.01, 2.04)*	1.46 (1.03, 2.09)*
Large	4.8		(Reference)	(Reference)
Average	5.0		0.88 (0.64, 1.20)	0.92 (0.67, 1.26)
Small	5.9		0.94 (0.62, 1.42)	1.01 (0.66, 1.53)
Very small	7.8		0.94 (0.66, 1.33)	0.99 (0.70, 1.41)
Child age (in months)				
≤12	18.3		(Reference)	(Reference)
12–24	3.6		0.16 (0.12, 0.21)*	0.16 (0.12, 0.21)*
>24	0.5		0.02 (0.01, 0.03)*	0.02 (0.01, 0.03)*
The plurality of a child				
Yes	20.4		5.57 (3.71, 8.36)*	5.41 (3.59, 8.16)*
No	5.4		(Reference)	(Reference)
Place of delivery				
Home	6.7		2.35 (1.81, 3.05)*	2.21 (1.69, 2.90)*
Institution	4.0			(Reference)
The place where food is cooked				
Outside the house	5.1			(Reference)
Inside but in a separate room	5.0			1.17 (0.72, 1.86)
Inside but not in a separate room	7.6			1.49 (1.17, 1.90)*
Type of residence				
Urban	3.1			(Reference)
Rural	6.5			1.64 (0.99, 2.72)
Region				
Tigray	3.8			(Reference)
Afar	8.7			0.78 (0.43, 1.43)
Amhara	4.8			0.79 (0.46, 1.34)
Oromia	5.5			1.11 (0.67, 1.84)
Somali	6.4			2.89 (1.48, 5.65)*
Benishangul	7.4			1.55 (0.92, 2.59)
SNNPR	5.4			1.22 (0.72, 2.06)
Gambela	6.1			1.36 (0.77, 2.38)
Harari	6.9			2.08 (1.12, 3.86)*
Addis Ababa	2.7			0.99 (0.44, 2.21)
Dire Dawa	5.5			2.21 (1.17, 4.18)*
Random effects				
ICC in %		**7.6**	**5.9**	**5.6**
Model comparison				
Log-likelihood		**−2130.6**	**−1581.8**	**−1566.2**

^a^*Ten-unit increase. Statistically significant at *p* < 0.05. **Adjusted for socio-demographic variables. ***Adjusted for socio-demographic and child-related variables. ****Adjusted for socio-demographic, child-related, environmental-related, and community level variable. Model 1 was built previously separately for each controlled variable.

Moreover, in the final model (Model 4), children under five with mothers without formal education (*p* = 0.02), very large birth size (*p* = 0.04), those who were twins (*p* < 0.001), those born at home (*p* < 0.001), those whose food was cooked inside a house without a separate room (*p* = 0.001), and residing in the Somali (*p* = 0.002), Harari (*p* = 0.02), and Dire Dawa (*p* = 0.02) regions were more likely to die, compared with those whose mothers had a secondary and above education, a large birth size, singletons, delivered in an institution, whose food was cooked outside of the house, and resided in the Tigray region, respectively; the results were statistically significant. In contrast, children under five with a family size above three (*p* < 0.001), were between 12 and 24 months (*p* < 0.001), age ≥ 24 months (*p* < 0.001), and were girls (*p* < 0.001) were less likely to die, compared with a family size ≤3, age ≤12, and being a boy under five, respectively; the results were statistically significant (Table 4).

## Discussion

This study assessed the relationship between long-term exposure to ambient fine particulate concentration levels and the mortality of children under five in Ethiopia. The study used a community-based two-stage survey design and satellite-based ambient PM_2.5_ exposure level. Total PM_2.5_ is significantly and positively associated with under-five mortality. It found that the annual mean ambient total PM_2.5_ children are exposed to is higher than the health-recommended concentration level. These findings revealed PM_2.5_ plays a significant role in the burden of under-five mortality. The results also provide insights for the government on the importance of increasing its effort in monitoring air quality to reduce its pollution level and negative effect on health.

The lifetime annual mean satellite-based ambient PM_2.5_ children are exposed to in their lifetime was 20.1 ± 3.3 μgm^−3^. This is beyond the World Health Organization (WHO) 2021 guideline—which updated the 2005 global air quality guideline—annual exposure limit of 5 μgm^−3^ (20). The result is consistent with evidence from LMICs such as China, India, Pakistan, Bangladesh (19), Africa, and Eastern Mediterranean (3) where almost no population, including children under five, breathe air below 10 μgm^−3^ PM_2.5_ concentration. Higher levels of PM_2.5_ is still a problem in European Member (EU) states too. According to the European Environment Agency (EEA), 97% of European urban residents are exposed to PM_2.5_ higher than 5 μgm^−3^. The road transport, energy, industry, and agriculture sectors are the sources of PM_2.5_ in Europe (41). In China and India, however, coal, construction dust, and burning solid fuels are the major emitters of PM_2.5_ (19, 23). In Ethiopia, most households—almost 70% in urban and 99% in rural areas—depend on solid fuels, mainly woody biomass, animal dung, and crop stalks (29). A study conducted on source apportionment of PM_2.5_ in Addis Ababa found that traffic flow, biomass, and dust sources are the major constituents (42).

Adjusting for other variables, a 10 μgm^−3^ increase in ambient PM_2.5_ was significantly associated with more odds of death. Furthermore, children in households whose food was cooked outside of the house, with mothers without formal education, who had a very large birth size, who were twins, and who were born at home were found to be significantly and positively associated with under-five mortality. On the contrary, children with a family size above three, between 12 and 24 months, age ≥24 months, and who were girls were significantly and negatively associated with under-five mortality.

A significant association between a 10 μgm^−3^ increase in ambient PM_2.5_ and under-five mortality that was found in this study is consistent with previous studies in India (43), Asia (44, 45), and sub-Saharan Africa (26). However, there is a difference between previous studies and this study’s estimated effect sizes; there was an increase in odds of under-five mortality with a 10-unit increase in lifetime annual mean PM_2.5_ concentration, adjusting for other variables (17, 46). This might be attributed to the use of different definitions—death per 1,000 live births—, separate estimations for the different types of PM_2.5_ (total PM_2.5_ versus salt and dust removed PM_2.5_), and differences in adjusted variables. It may also be because the estimate by Karimi and Shokrinezhad (46) was a pooled estimate, and the majority of studies included were from developed nations. The magnitude of the effect of PM_2.5_ on under-five mortality is higher among developing and least developed countries compared to developed countries (47). Fine particulate matter, as a result of its smaller mass concentration diameter size, can penetrate the blood-gas barrier. This allows the different chemical constituents of PM_2.5_ to circulate in the blood system and reach the different human organs (48). Especially, PM_2.5_ deposition in children is higher due to their fast breathing, lower nasal filtration, higher metabolic rate, and limited ability to metabolize toxic pollutants (4). Different substances, i.e., organic matter, elemental carbon, soil dust, sulfate, nitrate, and ammonium ions, form PM_2.5_ although it varies from place to place (49). Nephrotoxic heavy metals—chromium and cadmium—components of PM_2.5_ can also reach the kidney to affect its function (50), which might lead to death. PM_2.5_ is also associated with notable under-five mortality killer diseases pneumonia (3, 9, 14) and ARI (14, 15, 51).

Environmental-related variables—the type of drinking water source, toilet facility, and cooking fuel—were not significant in the final model. The following reasons could be the important factors that come into play. First, not all sources labeled as improved drinking water are free of fecal or disease-causing organisms (52). Second, there is a reintroduction of microbes into drinking water during collection by contaminated hands, containers, utensils, and/or insects (53, 54). Finally, households with improved drinking water may not necessarily have improved toilet facilities or vice versa (55). Even after access to improved facilities, the quality is influenced by human behavior, i.e., hand washing, personal hygiene, and consistent use of improved facilities (53, 54). Similarly, the use of clean fuel does not mean households are well-ventilated and the indoor air is relatively acceptable (56). This study supports the statement. Cooking food inside a house with no separate room, a proxy to ventilation, was associated with higher odds of under-five mortality.

## Strengths and limitation

The study provides insights into the relationship between ambient PM_2.5_ concentration level and under-five mortality at the individual level for the first time, and to the best of our knowledge, at the national level in Ethiopia. The study overcame the absence of ground-level ambient PM_2.5_ concentration data by using a freely available satellite-based estimate of ACAG.

The study is limited in accounting for indoor stay (PM_2.5_ exposure from indoor air was not quantified) and the movement of children from place to place in determining exposure to ambient PM_2.5_. It is also limited in not using ground-based PM_2.5_ nor making adjustments to the retrieved satellite-based PM_2.5_ as it is subject to measurement error. Currently, ground-based data is assumed to be a gold standard relative to satellite-based data. The authors compared the annual mean PM_2.5_ from satellite-based measurements and the three land-stationed BAM PM_2.5_ monitors in Addis Ababa (57) for similar geographical points. A large concentration difference was noticed at Tikur Anbessa Specialized Hospital and International Community School. However, a similar concentration level was observed at the United States (US) Embassy in Addis Ababa. A difference is expected considering that validated satellite-based PM_2.5_ has used a small number of land stations in Africa, including Ethiopia (58, 59). As a result, caution should be taken in interpreting the results of this study.

## Conclusion

This study determined the annual mean ambient PM_2.5_ exposure of children under five in their lifetime. Fine particulate matter annual exposure concentration among children under five exceeded the WHO recommended level. Ambient PM_2.5_ was significantly associated with higher odds of under-five mortality, adjusting for other variables. These results support the importance of taking measures to improve ambient air quality. The government should take strong actions to reduce air pollution and help achieve the goal set to decrease under-five mortality. The authors recommend further investigation which considers movement and indoor stay in determining ambient PM_2.5_ exposure in Ethiopia. It is also recommended to identify which constituents of PM_2.5_ are greatly affecting under-five mortality in Ethiopia.

## Data availability statement

Publicly available datasets were analyzed in this study. This data can be found at: https://dhsprogram.com/Data, https://sites.wustl.edu/acag/datasets/surface-pm2-5/.

## Ethics statement

The studies involving human participants were reviewed and approved by the College of Health Sciences of Addis Ababa University Ethical Review Committee. Written informed consent to participate in this study was provided by the participants’ legal guardian/next of kin.

## Author contributions

ABS did the study design, data acquisition, data cleaning, data analysis, and interpretation and contributed to the drafting, revising, and editing of this manuscript. AK and WT participated in the principal supervision, interpretation, and revision of the final manuscript. All authors read and approved the final manuscript.

## Conflict of interest

The authors declare that the research was conducted in the absence of any commercial or financial relationships that could be construed as a potential conflict of interest.

## Publisher’s note

All claims expressed in this article are solely those of the authors and do not necessarily represent those of their affiliated organizations, or those of the publisher, the editors and the reviewers. Any product that may be evaluated in this article, or claim that may be made by its manufacturer, is not guaranteed or endorsed by the publisher.

## Footnotes

^1^ https://dhsprogram.com/Data

^2^ https://washdata.org/monitoring/drinking-water

## Abbreviations

ACAG: Atmospheric Composition Analysis Group
AOD: Aerosol optical depth
AOR: Adjusted odds ratio
ARI: Acute respiratory illness
CI: Confidence interval
CRS: Coordinate Reference System
DHS: Demographic Health Survey
EA: Enumeration Area
EDHS: Ethiopian Demographic Health Survey
EEA: European Environment Agency
EU: European Union
GPS: Global Positioning System
ICC: Interclass correlation
LMICs: Low- and middle-income countries
PM: Particulate matter
PM_2.5_: Particulate matter mass concentration size below 2.5 μm diameter
SD: Standard deviation
ULRI: Upper and lower respiratory tract infection among children
US: United States
VIF: Variance inflation factor
WHO: World Health Organization

## References

[1] World Health Organization. Ambient air pollution: a global assessment of exposure and burden of disease. World Health Organization. (2016). Available at: https://apps.who.int/iris/handle/10665/250141

[2] Katoto PDMC Byamungu L Brand AS Mokaya J Strijdom H Goswami N. Ambient air pollution and health in sub-Saharan Africa: current evidence, perspectives and a call to action. Environ Res. (2019) 173:174–88. doi: 10.1016/j.envres.2019.03.029, PMID: 30913485

[3] World Health Organization. Air pollution and child health: prescribing clean air: summary. World Health Organization. (2018). Report No. WHO/CED/PHE/18.01. Available at: https://apps.who.int/iris/handle/10665/275545

[4] Saadeh R Klaunig J. Child’s development and respiratory system toxicity. J Environ Anal Toxicol. (2014) 4:233. doi: 10.4172/2161-0525.1000233

[5] Heinrich J Slama R. Fine particles, a major threat to children. Int J Hyg Environ Health. (2007) 210:617–22. doi: 10.1016/j.ijheh.2007.07.012, PMID: 17766181

[6] World Health Organization. Occupational and environmental health team. WHO air quality guidelines for particulate matter, ozone, nitrogen dioxide and sulfur dioxide: global update 2005: summary of risk assessment. World Health Organization. (2006). Report No. WHO/SDE/PHE/OEH/06.02. Available at: https://apps.who.int/iris/handle/10665/69477

[7] Ren L Yang W Bai Z. Characteristics of major air pollutants in China. Adv Exp Med Biol. (2017) 1017:7–26. doi: 10.1007/978-981-10-5657-4_2 29177957

[8] WHO/Europe. Air quality - health effects of particulate matter. Policy implications for countries in eastern Europe, Caucasus and Central Asia. (2013). Available at: https://www.euro.who.int/en/health-topics/environment-and-health/air-quality/publications/2013/health-effects-of-particulate-matter.-policy-implications-for-countries-in-eastern-europe,-caucasus-and-central-asia-2013

[9] Shi W Liu C Annesi-Maesano I Norback D Deng Q Huang C. Ambient PM2.5 and its chemical constituents on lifetime-ever pneumonia in Chinese children: a multi-center study. Environ Int. (2021) 146:106176. doi: 10.1016/j.envint.2020.106176, PMID: 33220537

[10] Darrow LA Klein M Flanders WD Mulholland JA Tolbert PE Strickland MJ. Air pollution and acute respiratory infections among children 0-4 years of age: an 18-year time-series study. Am J Epidemiol. (2014) 180:968–77. doi: 10.1093/aje/kwu234, PMID: 25324558 PMC4224364

[11] Zhang D Li Y Chen Q Jiang Y Chu C Ding Y. The relationship between air quality and respiratory pathogens among children in Suzhou City. Ital J Pediatr. (2019) 45:123. doi: 10.1186/s13052-019-0702-2 31547841 PMC6757402

[12] Liu Q Xu C Ji G Liu H Shao W Zhang C. Effect of exposure to ambient PM2.5 pollution on the risk of respiratory tract diseases: a meta-analysis of cohort studies. J Biomed Res. (2017) 31:130. doi: 10.7555/JBR.31.20160071 28808195 PMC5445216

[13] Perin J Mulick A Yeung D Villavicencio F Lopez G Strong KL. Global, regional, and national causes of under-5 mortality in 2000–19: an updated systematic analysis with implications for the sustainable development goals. Lancet Child Adolesc Health. (2022) 6:106–15. doi: 10.1016/S2352-4642(21)00311-4. 34800370 PMC8786667

[14] World Health Organization. Children: improving survival and well-being. (2020). Available at: https://www.who.int/news-room/fact-sheets/detail/children-reducing-mortality

[15] World Health Organization. Pneumonia: WHO fact sheet on pneumonia. (2021). Available at: https://www.who.int/news-room/fact-sheets/detail/pneumonia

[16] Egondi T Ettarh R Kyobutungi C Ng N Rocklöv J. Exposure to outdoor particles (PM2.5) and associated child morbidity and mortality in socially deprived neighborhoods of Nairobi, Kenya. Atmosphere. (2018) 9:351. doi: 10.3390/atmos9090351

[17] Owili PO Lien WH Muga MA Lin TH. The associations between types of ambient PM2.5 and under-five and maternal mortality in Africa. Int J Environ Res Public Health. (2017) 14:359. doi: 10.3390/ijerph14040359 28358348 PMC5409560

[18] Heft-Neal S Burney J Bendavid E Burke M. Robust relationship between air quality and infant mortality in Africa. Nature. (2018) 559:254–8. doi: 10.1038/s41586-018-0263-3, PMID: 29950722

[19] HEI. Health Effects Institute. State of global air 2019. Special report. Boston, MA: Health Effects Institute (2019) Available at: https://www.stateofglobalair.org/sites/default/files/soga_2019_report.pdf.

[20] World Health Organization. WHO global air quality guidelines: particulate matter (PM2.5 and PM10), ozone, nitrogen dioxide, sulfur dioxide and carbon monoxide World Health Organization (2021) Available at: https://apps.who.int/iris/handle/10665/345329. 34662007

[21] Liu L Oza S Hogan D Chu Y Perin J Zhu J. Global, regional, and national causes of under-5 mortality in 2000–15: an updated systematic analysis with implications for the sustainable development goals. Lancet. (2016) 388:3027–35. doi: 10.1016/S0140-6736(16)31593-8, PMID: 27839855 PMC5161777

[22] Wallner P Hutter HP Moshammer H. Worldwide associations between air quality and health end-points: are they meaningful? Int J Occup Med Environ Health. (2014) 27:716–21. doi: 10.2478/s13382-014-0305-5, PMID: 25209316

[23] Brauer M Freedman G Frostad J van Donkelaar A Martin RV Dentener F. Ambient air pollution exposure estimation for the global burden of disease 2013. Environ Sci Technol. (2016) 50:79–88. doi: 10.1021/acs.est.5b03709 26595236

[24] Atkinson RW Kang S Anderson HR Mills IC Walton HA. Epidemiological time series studies of PM2.5 and daily mortality and hospital admissions: a systematic review and meta-analysis. Thorax. (2014) 69:660–5. doi: 10.1136/thoraxjnl-2013-204492 24706041 PMC4078677

[25] Cui P Huang Y Han J Song F Chen K. Ambient particulate matter and lung cancer incidence and mortality: a meta-analysis of prospective studies. Eur J Pub Health. (2015) 25:324–9. doi: 10.1093/eurpub/cku145 25201901

[26] Bachwenkizi J Liu C Meng X Zhang L Wang W van Donkelaar A. Fine particulate matter constituents and infant mortality in Africa: a multicountry study. Environ Int. (2021) 156:106739. doi: 10.1016/j.envint.2021.106739 34217038

[27] Sharrow D Hug L You D Alkema L Black R Cousens S. Global, regional, and national trends in under-5 mortality between 1990 and 2019 with scenario-based projections until 2030: a systematic analysis by the UN inter-agency Group for Child Mortality Estimation. Lancet Glob Health. (2022) 10:e195–206. doi: 10.1016/S2214-109X(21)00515-5 35063111 PMC8789561

[28] United Nations Inter-agency Group for Child Mortality Estimation (UN IGME). Levels and Trends in child mortality: report 2020, estimates developed by the United Nations inter-agency Group for Child Mortality Estimation. New York, NY: United Nations Children’s Fund (2020) Available at: https://www.unicef.org/media/79371/file/UN-IGME-child-mortality-report-2020.pdf.pdf.

[29] EPHI, FMoH, ICF. Ethiopia mini demographic and health survey 2019. (2021). Available at: https://dhsprogram.com/publications/publication-FR363-DHS-Final-Reports.cfm

[30] CSA/Ethiopia CSA, ICF. Ethiopia demographic and health survey 2016. (2017). Available at: https://dhsprogram.com/publications/publication-fr328-dhs-final-reports.cfm

[31] Deribew A Tessema GA Deribe K Melaku YA Lakew Y Amare AT. Trends, causes, and risk factors of mortality among children under 5 in Ethiopia, 1990-2013: findings from the global burden of disease study 2013. Popul Health Metrics. (2016) 14:42. doi: 10.1186/s12963-016-0112-2, PMID: 27891065 PMC5109762

[32] WHO:GHO. Causes of child death. (2021). Available at: https://www.who.int/data/gho/data/themes/topics/indicator-groups/indicator-group-details/GHO/causes-of-child-death

[33] WorldPop. Where is Ethiopia in the world? (2021). Available at: https://worldpopulationreview.com/countries/ethiopia/location

[34] GEOHealth Hub. Report: environmental exposures, occupational safety, and climate change in Ethiopia. Eastern Africa GEOHealth Hub. (2015). Available at: https://geohealth-hub.org/planning-grant/reports/report-environmental-exposures-occupational-safety-and-climate-change-in-ethiopia/

[35] van Donkelaar A Martin RV Brauer M Hsu NC Kahn RA Levy RC. Global estimates of fine particulate matter using a combined geophysical-statistical method with information from satellites, models, and monitors. Environ Sci Technol. (2016) 50:3762–72. doi: 10.1021/acs.est.5b05833, PMID: 26953851

[36] Croft TN Marshall AMJ Allen CK. Guide to DHS statistics. Rockville, MD: ICF (2018) Available at: https://dhsprogram.com/pubs/pdf/DHSG1/Guide_to_DHS_Statistics_DHS-7.pdf.

[37] Thabane L Mbuagbaw L Zhang S Samaan Z Marcucci M Ye C. A tutorial on sensitivity analyses in clinical trials: the what, why, when and how. BMC Med Res Methodol. (2013) 13:92. doi: 10.1186/1471-2288-13-92 23855337 PMC3720188

[38] Finch W Bolin J Kelley K. Multilevel modelling using R. Muncie, IN: Ball State University (2014).

[39] Senaviratna NAMR Cooray TMJA. Diagnosing multicollinearity of logistic regression model. Asian J Probab Stat. (2019) 5:1–9. doi: 10.9734/AJPAS/2019/v5i230132

[40] Bivand R Pebesma E Gómez-Rubio V. Applied spatial data analysis with R. New York, NY: Springer International Publishing (2013).

[41] European Environment Agency (EEA) Air quality in Europe 2021. European Environmental Agency, (2021).

[42] Tefera W Kumie A Berhane K Gilliland F Lai A Sricharoenvech P. Source apportionment of fine organic particulate matter (PM2.5) in Central Addis Ababa, Ethiopia. Int J Environ Res Public Health. (2021) 18:11608. doi: 10.3390/ijerph182111608, PMID: 34770121 PMC8583055

[43] Saraswat Y Bansal S. Health effects of sustained exposure to fine particulate matter: Evidence from India. Rochester, NY: Social Science Research Network (2020) Available at: https://papers.ssrn.com/abstract=3542183.

[44] Anwar A Ullah I Younis M Flahault A. Impact of air pollution (PM2.5) on child mortality: evidence from sixteen Asian countries. Int J Environ Res Public Health. (2021) 18:6375. doi: 10.3390/ijerph18126375, PMID: 34204659 PMC8296171

[45] Lien WH Owili PO Muga MA Lin TH. Ambient particulate matter exposure and under-five and maternal deaths in Asia. Int J Environ Res Public Health. (2019) 16:3855. doi: 10.3390/ijerph16203855 31614721 PMC6843620

[46] Karimi B Shokrinezhad B. Air pollution and mortality among infant and children under five years: a systematic review and meta-analysis. Atmospheric Pollut Res. (2020) 11:61–70. doi: 10.1016/j.apr.2020.02.006

[47] Liu S Wei Q Failler P Lan H. Fine particulate air pollution, public service, and under-five mortality: a cross-country empirical study. Healthcare. (2020) 8:271. doi: 10.3390/healthcare8030271 32823932 PMC7551449

[48] Xu W Wang S Jiang L Sun X Wang N Liu X. The influence of PM2.5 exposure on kidney diseases. Hum Exp Toxicol. (2022) 41:09603271211069982. doi: 10.1177/09603271211069982 35174736

[49] Tefera W Kumie A Berhane K Gilliland F Lai A Sricharoenvech P. Chemical characterization and seasonality of ambient particles (PM2.5) in the city Centre of Addis Ababa. Int J Environ Res Public Health. (2020) 17:6998. doi: 10.3390/ijerph17196998, PMID: 32987918 PMC7579520

[50] Wedeen RP Quian L. Chromium-induced kidney disease on JSTOR. (1991). Available at: https://www.jstor.org/stable/3431139 10.1289/ehp.92-1519395 PMC1519395 1935854

[51] Abudureyimu K Suryadhi MAH Yorifuji T Tsuda T. Exposure to fine particulate matter and acute upper- and lower-respiratory tract infections (AURI and ALRI) in children under five years of age in India. Arch Environ Occup Health. (2022) 78:1–6. doi: 10.1080/19338244.2022.2047584 35285781

[52] World Health Organization. Rapid assessment of drinking-water quality: a handbook for implementation. (2012). Available at: https://www.who.int/publications-detail-redirect/789241504683

[53] Kumie A. The effect of improved water and sanitation on diarrhea: evidence from pooled Ethiopia demographic and health surveys – a multilevel mixed-effects analysis. Ethiop J Health Dev. (2020) 34:268–276.

[54] Shaheed A Orgill J Montgomery MA Jeuland MA Brown J. Why improved water sources are not always safe. Bull World Health Organ. (2014) 92:283–9. doi: 10.2471/BLT.13.119594 24700996 PMC3967570

[55] Andualem Z Dagne H Azene ZN Taddese AA Dagnew B Fisseha R. Yeshaw Y households access to improved drinking water sources and toilet facilities in Ethiopia: a multilevel analysis based on 2016 Ethiopian demographic and health survey. BMJ Open. (2021) 11:e042071. doi: 10.1136/bmjopen-2020-042071 PMC7978246 33737423

[56] Sanbata H Asfaw A Kumie A. Association of biomass fuel use with acute respiratory infections among under- five children in a slum urban of Addis Ababa, Ethiopia. BMC Public Health. (2014) 14:–1122. doi: 10.1186/1471-2458-14-1122 PMC4237768 25358245

[57] Kumie A Worku A Tazu Z Tefera W Asfaw A Boja G. Fine particulate pollution concentration in Addis Ababa exceeds the WHO guideline value: results of 3 years of continuous monitoring and health impact assessment. Environ Epidemiol. (2021) 5:e155. doi: 10.1097/EE9.0000000000000155, PMID: 34131616 PMC8196089

[58] van Donkelaar A Hammer MS Bindle L Brauer M Brook JR Garay MJ. Monthly global estimates of fine particulate matter and their uncertainty. Environ Sci Technol. (2021) 55:15287–300. doi: 10.1021/acs.est.1c05309 34724610

[59] Hammer MS van Donkelaar A Li C Lyapustin A Sayer AM Hsu NC. Global estimates and long-term trends of fine particulate matter concentrations (1998–2018). Environ Sci Technol. (2020) 54:7879–90. doi: 10.1021/acs.est.0c01764 32491847

